# Therapists’ Experience of Video Consultation in Specialized Mental Health Services During the COVID-19 Pandemic: Qualitative Interview Study

**DOI:** 10.2196/23150

**Published:** 2021-07-15

**Authors:** Monika K Gullslett, Eli Kristiansen, Etty R Nilsen

**Affiliations:** 1 Norwegian Center for E-Health Research University Hospital of North Norway Tromsø Norway; 2 School of Business and Economics University of Tromsø (UiT) Alta Norway

**Keywords:** mental health services, recovery, telemedicine, telepsychiatry, video consultation, COVID-19, coronavirus, therapists’ experiences, hospital services

## Abstract

**Background:**

As part of political and professional development with increased focus on including service users within mental health services, these services are being transformed. Specifically, they are shifting from institutional to noninstitutional care provision with increased integration of the use of electronic health and digitalization. In the period from March to May 2020, COVID-19 restrictions forced rapid changes in the organization and provision of mental health services through the increased use of digital solutions in therapy.

**Objective:**

The aim of this study was to develop and advance comprehensive knowledge about how therapists experience the use of video consultation (VC). To reach this objective, we evaluated therapists’ experiences of using VC in specialized mental health services in the early phase of COVID-19 restrictions. The following questions were explored through interviews: Which opportunities and challenges appeared when using VC during the period of COVID-19 restrictions? In a short-term care pathway, for whom does VC work and for whom does it not work?

**Methods:**

This study employed a qualitative approach based on an abductive strategy and hermeneutic-phenomenological methodology. Therapists and managers in mental health departments in a hospital were interviewed via Skype for Business from March to May 2020, using a thematic interview guide that aimed to encourage reflections on the use of VC during COVID-19 restrictions.

**Results:**

Therapists included in this study experienced advantages in using VC under circumstances that did not permit face-to-face consultations. The continuity that VC offered the service users was seen as a valuable asset. Various negative aspects concerning the therapeutic environment such as lack of safety for the most vulnerable service users and topics deemed unsuitable for VC lowered the therapists’ overall impression of the service. The themes that arose in the data analysis have been categorized in the following main topics: (1) VC—“it’s better than nothing”; (2) VC affects therapists’ work situation—opportunities and challenges in working conditions; and (3) challenges of VC when performing professional assessment and therapy on the screen.

**Conclusions:**

Experiences with VC in a mental health hospital during COVID-19 restrictions indicate that there are overall advantages to using VC when circumstances do not permit face-to-face consultations. Nevertheless, various negative aspects in the use of VC lowered the therapists’ overall impression of VC. Further qualitative research is needed, and future studies should focus on service users’ experiences, cocreation between different stakeholders, and how to scale up the use of VC while ensuring that the service provided is appropriate, safe, and available.

## Introduction

### Research Context

Mental health service provision in Norway is changing, and there is an increase in noninstitutional care provision for people with long-term mental health problems [1]. One central area of attention is the provision of follow-up care at a distance, including the use of electronic consultations and video consultations (VC) within mental health services in hospitals, which has become more common in recent years [2,3]. Nevertheless, implementation of VC has been slow [4,5]. However, in the period from March to May of 2020, the COVID-19 restrictions enforced a radical change in how health care services were organized. A need to find alternative solutions to face-to-face consultations emerged to enable safe treatment of service users without risk of contagion. The use of VC in different parts of the health care service increased; as such, during the first period of restrictions due to COVID-19, therapists and service users were forced to use technology to communicate, whether or not they had experience with this kind of technology. The pandemic thus became a magnifying glass, revealing both challenges and advantages in the use of VC. This increased use of VC raises questions concerning how the technology may affect both the quality and availability of services in mental health, especially with regard to following up with those in recovery and in need of complex and long-term services.

Reasons for seeking help from mental health services are often based on negative experiences in relationships and difficulties in coping with everyday life. There is a need to tailor digital services to promote recovery and change in service users’ mental health state, and to support and improve social relations and coping strategies in the context of everyday life [6,7]. Along the continuum of service provision, VC may be used as part of follow-up care in certain phases of the helping process. In some cases, VC can offer new opportunities for understanding and treating mental health experiences in context. This, in turn, can lead to a greater emphasis on psychosocial approaches, involving service users and carers as active partners in care provision, and refocusing outcomes of services to align with daily life, employment, and other aspects of social inclusion. By contrast, the use of VC may prove challenging for therapists when they seek a comprehensive understanding of the service user’s complex situation within their individual context. A final, but equally important, question that has emerged alongside the increased use of VC concerns the impact it has on therapists’ work situation [8] and how they cope with the technology in their therapeutic relationships. 

### Background

Norway’s national health and hospital plan [1] emphasizes the goal of realizing a sustainable health care service based on each service user’s needs at all levels of the service provided. Both in the meeting between the service user and the therapist, and in the development of the health and care services, the vulnerable voice of the service user must be heard. As part of political and professional development along with an increased focus on including service users within mental health services, services are being transformed; specifically, they are shifting from institutional to noninstitutional care provision with increased use of electronic health (eHealth) and digitalization [1]. Integrating video as a consultation platform is part of the innovation strategy described in Norway’s latest national health and hospital plan [1].

The use of VC underscores a shift in the focus of care from treating service users in hospital departments to flexible models within the continuum of care. This change is aimed at increasing the focus on recovery-oriented services [5,9]. Recovery in mental health is a concept that has had a range of definitions over the past several decades [10,11]. The concept is used both to describe an approach and as the process the individual goes through to improve their mental health. These two definitions are interlinked in that recovery as an approach has developed from being described as an individual process [12]; currently, the inclusion of social recovery, and relational and contextual factors are emphasized [13,14]. Being in recovery from severe mental health illness is often a complex process that involves a range of stakeholders, including both professionals and peer support [14-16], and it is often described as a process in which the service user is in the driver’s seat for their own recovery to live a self-directed life [11]. In this expanded view of recovery, digital solutions such as VC may have an impact on the process of recovery in numerous ways [9]. The recovery tradition also emphasizes the service user as a human being and not as a diagnosis [10,17]; moreover, in this tradition, the use of language is seen as an important tool to empower individuals with mental health problems, and especially to reduce stigma [18,19]. The term “service user” rather than “patient” is used to refer to people in treatment for mental health problems. “Patient” is a defined role and a theoretical construct closely linked to a medical perspective, and it is a concept used to define, both legally and professionally, an individual receiving any kind of health service.

The use of communication technologies and tools in assessment and therapy in mental health services is not a new phenomenon [4], and there are many different terms used to designate digital treatment, including “telehealth,” “telepsychiatry,” “telepsychology,” “eHealth,” “telemedicine,” and “video consultations” [4]. In this paper, we use “VC” to refer to an online meeting between a therapist and a service user. There is also a substantial body of research on the use of telehealth in mental care. A recent review of the field of telepsychiatry highlights the use of digital solutions as an effective way to improve access, enhance quality, and provide efficient care [4,20]. VC contributes to the provision of services in the service user’s home or other local settings, which may empower the service user, incorporate their voice, and contextualize their mental health problems as part of their everyday life. This promotes the opportunity for the service user to participate in their own recovery process, which is considered important from a recovery perspective [4,7,21,22]. A crisis assessment study on the use of VC in acute mental care in Norway underscores the opportunity to provide continuous follow-up care for people in acute crisis, despite geographical distances and lack of psychiatrists in certain regions [23]. Use of VC between the therapists and the service user strengthened the involvement of the service user during the crisis assessment; specifically, it reduced uncertainty, created shared responsibility for decisions, and functioned as a safety net, even when the use of VC was not required [23-25]. Other recent studies on the use of telecare indicate that follow-up treatment at a distance for people with different diagnoses and backgrounds is effective and safe; these studies included both elderly people with depression and veterans in recovery from posttraumatic stress syndrome [3,26-29]. With regard to the former group, use of VC in therapy with elderly people suggests that VC supports mental health practice, especially as a useful alternative when face-to-face therapy is not possible [30]. Initial skepticism often disappears once the VC is experienced in action; any residual challenges seem to be related to technical problems and a lack of support from staff [31,32].

In general, findings from the above-mentioned studies indicate that the use of VC in treatment may be an efficient way to provide therapy. However, several of these were pilot studies, in which the implementation occurred in a limited area of the service with selected service users and therapists. We know that this kind of approach can lead to self-selection bias, as pilot studies often attract digitally optimistic and mature participants [32]. There can thus be a mismatch between pilot studies and real-world implementation; indeed, when the service has been implemented within the daily operations of a hospital, additional challenges have been revealed [32]. A study on experiences of VC implementation within the everyday operation of a mental health hospital is therefore essential, which will offer new knowledge for the field. Extant research also shows that from the service users’ perspective, both lack of face-to-face contact and technical challenges were seen as barriers [8,33], whereas from the health care providers’ perspective, physical presence and reading of subtle signs are central in high-quality care [34]. To date, few qualitative studies have dealt with in-depth experiences with the use of VC and recovery in mental health that included both therapists and service users. Given this gap, this study focused on therapists’ experiences and addressed the following research questions: Which opportunities and challenges appeared when using VC during the period of COVID-19 restrictions? In a short-term care pathway, for whom does VC work and for whom does it not work? 

### Case Context

This study was performed at a hospital located in northern Norway, which is a sparsely populated rural area. As the distances between service users and the hospital can be considerable, the hospital has worked to implement technologies for distance communication for decades. The VC system in use during the study period was Skype for Business, which the hospital had been using in this capacity for 3 years. Some of the therapists in the hospital’s mental health departments were experienced users of telecare and VC, both in terms of professional collaboration and therapy, with steady use over several years. This characteristic, however, does not apply to all of the therapists in the region. The data extracted from the electronic patient journal (EPJ) system show that the number of consultations in mental health care performed over video has been low, but the use has seen a slow annual increase in recent years. In 2017, 1% of all consultations were performed using video. In 2019, this number increased to 4%, and during the COVID-19 restrictions, the use of VC saw a dramatic increase. On March 12, 2020, Norway completely locked down, which meant that all public institutions—including schools, kindergartens, and offices—closed. During this period (March 15-30, 2020), 72% of all of consultations were performed over video. Owing to a decreasing rate of infection, the restrictions were slowly relaxed in mid-April; however, several restrictions remained in place [35]. Taking local levels of infection into consideration, permission to perform face-to-face consultations (with multiple safeguards in place regarding infection control) was granted. The use of VC thus slowly diminished once again, comprising only 21% of all consultations in the last part of May 2020 (Table 1).

**Table 1:** Use of video consultations in the hospital.

Period	Performed video consultations, n (%)	Performed face-to-face consultations, n	Total consultations, N
2017	178 (0.7)	27,563	27,741
2018	956 (3.2)	28,569	29,525
2019	1188 (4.1)	28,032	29,220
January 1 to March 15, 2020	341 (5.4)	5984	6325
March 16-30, 2020	352 (72)	139	491
April 2020	718 (58)	524	1242
May 2020	463 (21)	1748	2211

## Methods

### Research Design

A qualitative, explorative study using in-depth interviews was performed in a mental health hospital. The methodological approach was based in the social sciences, using an abductive strategy that aimed to uncover—and then interpret—knowledge about the social actors in question [36]. This entailed investigating how the therapists experienced, understood, and created a context for using VC in therapeutic meetings with their service users. This perspective worked well with the hermeneutic-phenomenological approach we employed in our analysis; moreover, our choice of research strategy was integrated into the objectives of the study and the research questions under investigation. For the purposes of this study, a hermeneutic-phenomenological perspective meant that the researchers sought an in-depth understanding of the participants’ real-world experiences around the use of VC during COVID-19 restrictions [37,38]. Further, the researchers’ own hermeneutic position entailed acknowledging that although the data collection and analysis were undertaken with a reflexive and open-minded view, the theoretical approach and researchers’ preconceptions would also affect the results.

### Interviews

In-depth interviews following a semistructured interview guide were conducted in late March to mid-May 2020, 2 weeks after the COVID-19 restrictions were introduced in Norway. The interviews were conducted on video. The first author (MG) conducted all interviews, and opened each interview by asking the therapist to tell a story about when, how, and why they had implemented VC in their mental health service for the first time. An interview guide was developed beforehand with the aim of mapping the implementation and use of VC from different perspectives; this guide was sent out to all informants prior to the interview. The interview guide was primarily used as a checklist as the interview progressed into more of a conversation. The interviewer was mindful of the fact that conducting interviews in this way may lead to a different information flow than that occurring in a face-to-face meeting, and that while the main objective of the abductive research strategy is to gain in-depth understanding of each participant’s perceptions, the use of digital tools may (negatively or positively) affect the process.

### Selection and Sample

When the COVID-19 restrictions were implemented, one of the recommendations for mental health workers was to follow up with service users by using VC [39]. A qualitative study had already been planned at the hospital on different aspects regarding the organization and implementation of VC during normal circumstances. When the societal lockdown occurred, we decided to accelerate the process to investigate the therapists’ experiences of being rushed into a large-scale implementation of VC in the hospital environment. We sent a request for participation to the management at the hospital on March 20, 2020. The management redistributed the request to everyone in the mental health departments, stating that participation should be given priority.

A total of 14 participants from different disciplines and departments were recruited. The participants worked with adults, adolescents, and children, in addition to performing family therapy; 13 were therapists and 1 was a department head. There was diversity in age, gender, and professional background among the informants: the youngest was 27 and the oldest was 66 years old at the time of the interviews, and there were 3 men and 11 women, 5 of whom had 6 or more years of education, whereas the rest had 3 or more years of experience. In this context, “therapists” is used to denote mental health professionals who are trained to provide treatments in different ways; as such, in this study, the therapists were psychiatrists, psychologists, nurses, and social workers with at least 3 years of university education.

### Analysis

All interviews were recorded and transcribed verbatim. The transcriptions were undertaken by a professional firm just after the interviews were completed. To validate the content, the first author read all of the transcriptions and compared them to the recorded interviews. The analysis was performed through a reflexive, open-minded, and abductive process, which enabled an intuitive understanding of the meaning of the text as a whole [36]. Following the initial in-depth reading of the interviews, the content was categorized and grouped together to identify important themes according to the research questions. The themes in the analysis arose through an iterative process of reading and interpreting to identify meaningful units [36-38]. 

### Ethics Approval and Considerations

The study was approved in advance by the ethical committee (PVO) at Helse Nord (project ID 2462). The participants were given both written and verbal information about the study before agreeing to participate. The included informants sent their consent forms via mail to the first author, which were stored without any connection to the gathered data material.

## Results

### Main Themes

Data were analyzed and categorized with regard to the research questions: Which opportunities and challenges appeared when using VC during the period of COVID-19 restrictions? In a short-term care pathway, for whom does VC work and for whom does it not work? According to the therapists, being forced to initiate the use of VC during COVID-19 restrictions to follow up with service users resulted in both positive and negative experiences. They also expressed an overall perception that the video format offered a necessary opportunity to maintain contact with service users during a challenging and abnormal period. However, several challenges were introduced when the VC was implemented, including the low quality of certain technological aspects, insecurity related to communicating on video, and challenges in managing the service users’ reluctance to participate in VC. The themes that arose during data analysis were categorized into three main topics: (1) VC—“it’s better than nothing”; (2) VC affects therapists’ work situation—opportunities and challenges in working conditions; and (3) challenges of VC when performing professional assessment and therapy on screen.

### VC—“It’s Better Than Nothing”

#### VC Promotes Continuity and Access to Service

As indicated by the analysis of data extracted from the EPJ system (see Table 1), the use of VC skyrocketed in the period immediately following the introduction of COVID-19 restrictions. This was also noted by one of the therapists interviewed: “I haven’t counted, but I can bet that as many as 85% to 90% of the consultations in the last 2 weeks were on Skype.” Conducting consultations on video allowed the treatment to continue despite the societal lockdown. For some service users, this was valuable; however, the therapists described several service users as reluctant to participate in VC, preferring to wait for the restrictions to ease to continue regular face-to-face treatment instead. From the therapists’ point of view, it was emphasized that VC allowed for closer follow-up and continuity in the treatment of the service users during the COVID-19 lockdown. In some cases, they found it important to encourage service users who were skeptical about the video format to participate in VC to secure continuity and enable follow-up care regarding potentially serious mental health problems. Further, the therapists reported that VC made it easier for service users with social anxiety to take part in consultations, similar to the benefits for users with mobility disabilities or those who worked offshore. They also emphasized how continuity is important when following up with service users with suicidal thoughts; here, a key element is scheduling future appointments to which the service user can look forward, and VC made this possible during the lockdown. Despite these positives, the therapists felt that the quality of the service was affected by the video format. One of the therapists described this challenge as follows:

It’s like baking your favorite cake with artificial sweeteners instead of sugar—it will work, and it tastes and looks okay, but there is something missing, it’s not the same quality. However, it’s definitely better than nothing.

#### Establishing and Maintaining a Relationship on Video

Initiating a therapeutic relationship on video can be challenging for both the service user and the therapist. The first conversation on video was described as generally consisting of an introduction to the service user’s progress plan, or, in some cases, a risk assessment concerning the severity of the service user’s suicide risk. The latter was found to be especially difficult to achieve on video. Meeting new service users on video could cause insecurity on both sides of the screen and, as one therapist mentioned, although it is always necessary to ensure that confidence and balance are established in the relationship, this is especially important when the initial consultation is on video. Indeed, one of the informants explained that if the therapist feels insecure with the video format, this can affect the power relations between the service user and the therapist. Another therapist felt it was important to provide information to the service user about how to communicate on VC and explain how the pathway of recovery would be addressed on video. Overall, the therapists agreed that it is preferable to meet the service users for the first time face to face to establish a good relationship, and that this would help make future consultations on video less scary and more productive. When employed in consultations with service users they already knew, the therapists felt that video could be a useful tool:

Yes, it was a new [service user] and we had not been able to meet physically, so we had the first consultation on Skype. This was a person I knew in advance and [the service user] also knew who I was, so we were not totally strangers to each other. It worked fine.

It should also be noted that some therapists did experience positive first meetings on video with new service users, although it helped when the therapist and the service user already knew one another. As one of the therapists stated: “I did not complete my education in psychology to meet people on a screen. I want to see them face to face.”

Some of the therapists found VC involving children and adolescents to be particularly challenging, as these service users could experience meeting the therapist on video as frightening in the absence of the natural human comfort and security a face-to-face meeting can provide:

Today we had a little 3-year-old who wanted to see us, but then she didn’t dare. “Oh no, I don’t dare,” she said. We had a very good conversation with the parents, but it can be a challenge for youngsters to join.

Nevertheless, therapists also reported positive experiences, in which children felt safe in the video conversations because the video format allowed them to be in their own home. Some adolescents were quite familiar with the video and internet format, and felt that they could control it—and were more in control when using it—regarding what to choose to display and present on video. However, given the above experiences, some of the therapists were surprised to find that some adolescent service users avoided VC. One possibility to explain this presented was that if it was the service users’ family who wanted them to receive treatment, the service users may have been using reluctance toward VC as an excuse to avoid therapeutic consultations. Nevertheless, the therapists highlighted that just because adolescent service users may be in a digitally mature age group and are used to online communication among themselves on social media, this does not necessarily imply a positive attitude toward VC:

I don’t know if it’s about talking to a professional or having some kind of treatment, that makes it difficult? I used to do phone calls, too, but it was hard to get mentally close and open up in the conversation. And maybe when you meet face to face you know better how to get into the right topics, maybe? I do not know.

With regard to family treatment, several of the therapists raised concerns about using video in consultations, as the focus in this kind of therapy is on creating a relationship with the service user (child) in their own home and monitoring the interaction between the child and the parents. The natural situation is difficult to observe on video, and the therapists feared that important aspects of the children’s behaviors were not displayed accurately on screen. The parental guidance consultations, in which only the parents are being guided by the therapist, and the interaction aspect (although beyond the scope of this study) were pointed to by therapists as working well on video.

### VC Affects Therapists’ Work Situation—Opportunities and Challenges in Working Conditions

#### Barriers to Effective Communication

On the one hand, working together using video provides an opportunity for closer follow-up and more flexibility in meetings both with service users and with colleagues, including collaborating with providers in other services. On the other hand, therapists also felt that their working conditions were negatively impacted by the video format, and that the communication with the service users changed when it occurred on the screen; as such, they described finding it difficult to make clinical judgments and experienced insecurity regarding the service users’ conditions. Several of the therapists revealed that they became exhausted and frustrated when performing VC for an entire workday and that communicating through the screen required a different kind of presence than face-to-face meetings.

You get pretty dizzy in your head when you talk to people on Skype. Some conversations last up to, erm, on average, it can last for an hour. But it depends on where you are in the course of treatment. I think you get tired in a different way in, in your head, when you have spent all day on the screen, sitting and talking like that.

The therapist quoted above had thought it might be easier to conduct consultations on the screen, as not being in the same room could eliminate the potential influence of emotions from the service user. Other therapists also elaborated on the differences between face-to-face and video consultations. One therapist mentioned:

If we look at the amount [of VC], it would have been really okay to have some consultations face to face to get variety. When all consultations were on video...well, I don’t know how to describe it. It is uncomfortable and it doesn’t feel like a good way to work.

During face-to-face consultations, small breaks often appear naturally during the conversation, and a break while one or the other is thinking feels safe and leads perhaps to a necessary pause in the conversation. In consultations on the screen, these small breaks can feel unnatural. As one therapist noted,

The contact feels a little reduced, a little more strained. You sit there staring. It is a deadlocked situation and it is difficult to take breaks. Breaks in the conversation quickly become unpleasant.

However, another informant pointed out that by working continuously on the screen, more experience with the format was gained, and this led to more natural conversations when using video; this therapist described that a natural approach to working with video developed over time, making it easier to interact in this specific format.

#### Coping With Technology

Technical problems were reported as severely affecting the quality and safety of VC. One therapist who had experienced a VC in which there were numerous technical problems described the consultation as highly unsuccessful. After the consultation, this therapist felt it necessary to apologize to the service user for the poor quality of the video and the fact that they had been unable to cope with the technology.

It’s a pretty bad start when you haven’t talked to this person before, like the [VC] I mentioned, and we have to give up the consultation for technical problems. We were about to have a first consultation and then we lost 10 to 15 minutes before we found out that it did not work. What impressions are you left with then, [as a service user]? I really wonder how it was for her the first time. I wasn’t very happy after that session.

In retrospect, the therapist regrets not testing the technology before the consultation, saying: “It’s our responsibility, isn’t it? We offer a type of counseling and then we mess it up or it works badly. It is our responsibility.” This therapist was thus left feeling insufficient, that the consultation was unprofessional, and that the VC left both the therapist and the service user feeling negatively about the experience. Distortions in the picture on the screen, disruptions in the sound, losing connection, and other technological interruptions were also felt to have potentially affected the emotional connection and interrupted the flow of a vulnerable conversation. As one therapist explained:

Yes, I try, but I don’t always know where the problem lies. I am not very good with technology, so…We had a case where we had to do it over the phone. I told the [service user] that she should get help from her partner the next time, and then it worked. While with another [service user] we gave up simply because we couldn’t make [the technology] work.

VC does require a good internet connection, which not all service users and therapists have at home. When technical problems occurred, the therapists told us that their solution was to call the service user via telephone. Some therapists said they tested the technology with other colleagues before conducting the initial conversation with service users. In this way, they avoided unfavorable situations and reduced their fears of using VC. The therapists also found it essential to ensure that the service user had their technology in order and felt comfortable using it. They felt that, as professionals, they had to offer any necessary help:

For [service users] who find video technology unfamiliar and difficult to use, they can experience it as a personal failure not to master the technology. They may place the blame for the technical problems on their own incompetence, and not on the different aspects of the technology or system failures.

One informant explained that if they felt insecure about the technology, they would be fully open about it to the service user to create balance in the relationship. This would also ensure that the service user would not feel like they were to blame for the problems with the technology.

Yes, I think it is safe to say it like it is. Maybe also be a bit humble and say that you are not entirely sure of the technology yourself. There may be some connection issues, but we will solve that by calling or doing a trial round first to see if we get it right. Then we can schedule a time for a conversation maybe the next day.

#### Lack of Transparency: Not Knowing Who is in the Room

When a service user was in a controlling or abusive relationship, therapists found it challenging to not meet face to face in the office. The therapists explained that it could be difficult to assess with certainty whether the service user could speak openly about how they really felt and was being treated, as the person responsible for the abuse could be in the room with the service user, but off screen. The controlling or abusive partner or parent may also have the opportunity to instruct the service user on what to tell the therapist, and the therapist has no way of knowing whether the service user is being observed during the consultation. As one therapist explained:

Her partner has demanded that the conversation take place in a room that he has access to. So, when taking care of [service users] who have manipulative, controlling partners, Skype and telephone represent something I cannot handle. I also have to consider what I say to her [the service user]. If there is something she has told me when we were alone, then I cannot begin the consultation by saying, “The last time, you told me that your partner hit you.” He might be sitting right there, you know.

According to this therapist, for some service users, abuse is embedded in their everyday life, providing a clear limitation regarding what therapists may be comfortable addressing in a VC. This then leads us to the next theme, which is performing therapy on the screen.

### Challenges of VC When Performing On-Screen Professional Assessment and Therapy

#### Suitable and Unsuitable Topics When Using Video

The therapists reported that some conditions and moods were challenging to detect through the video camera, as both body language and other nonvisual impressions disappear. Serious diagnoses and psychological investigations were mentioned as particularly difficult to conduct and discuss over video. Indeed, distrust in the technology and doubt that the VC would progress without disruptions kept many of the therapists from pursuing the most sensitive themes and subjects. They feared that the video connection would break down in the middle of a critical conversation and wanted to avoid having to ask a service user to repeat part of a longitudinal trauma monologue. The most traumatic incidents could be difficult to discuss on video for fear of technical problems or not having control over the service user’s environment. Consequently, among other reasons, the therapists did not find video to be a suitable medium for discussing service users’ most vulnerable feelings, nor was it easy to find the balance between keeping the therapy moving forward and not digging too deeply into the service users’ most vulnerable feelings or traumas. Closing the consultations also represented a potential challenge:

I think this is an important aspect, because I have no control after they leave my office. When I meet the person face to face, I have more control over my assessment of what state they are in when they leave.

One concern shared by several of the therapists centered around the challenge of knowing whether service users were left in an unresolved state and closing the consultation in an appropriate way can be challenging on the screen. A VC can be ended more abruptly than an office visit, by simply pressing the “off” button at the end of the consultation. The therapists feared that ending the conversation too rapidly could be harmful, especially if the consultation had dealt with traumatic subjects. By contrast, avoiding a long journey home by car after an emotional consultation was mentioned as a positive feature with VC, especially for service users with a commute of several hours.

#### VC as a Filter for Emotions and Health Conditions

Several of the therapists experienced that the video format created distance, which in turn felt like a filter or an obstacle with regard to obtaining relevant information about the service user’s condition. According to one therapist:

It gets…VC becomes like a filter between us, which, in a way maybe is more apparent on video than normally [face-to-face consultation] (…) The biggest difference is the challenge of simply understanding the nonverbal communication.

Nonverbal communication that is harder to detect on video might be a glance, a short break from the conversation while looking away, small body movements, jittery fiddling, and similar, almost invisible, movements that although sometimes hard to notice are important for the therapist’s assessment of the service user’s mental health condition. One therapist described this challenge as follows:

I am not sure how to explain it exactly, because it depends on how observant you are. You notice little things. I can listen to and observe a lot when people talk…perceive things.

This aspect of VC, in which the therapist loses information through the digital filter, was cited as the most challenging and risky part of performing consultations on the screen. One therapist had received a referral stating that a service user had a specific smell; however, because the consultation was performed over video, the therapist lost the opportunity to smell and experience the service user. The therapist explained: “There may be something about cleanliness and, what can I say, if a person does not take care of himself it can be a sign of, for example, depression.” With the digital filter in place, there is thus a risk of losing important information regarding certain health conditions, elements, and aspects that may be crucial to the therapist’s ability to see the whole picture. In complex situations, VC did not feel like a safe alternative because of this filter and affected the therapists’ ability to make clinical judgments about the service users’ conditions. Investigating the condition of the service user through the use of standard tools, especially validated schemes to generate diagnoses, was also mentioned as challenging. Indeed, procedures such as these were largely put on hold by therapists until it was possible to meet face-to-face.

### Summary of Themes

To summarize the findings, Table 2 includes the main themes and subthemes that emerged in the analysis.

**Table 2:** Summary of themes and subthemes related to video consultations (VC).

Theme	Subthemes
VC—“it’s better than nothing”	VC promotes continuity and access to services; establishing and maintaining relationships on video
VC affects therapists’ work situation—opportunities and challenges in working conditions	Coping with technology; lack of transparency—not knowing who is in the room
Challenges of VC when performing professional assessment and therapy on screen	Suitable and unsuitable topics when using video; VC as a filter for emotions and health conditions

## Discussion

### Principal Findings

In the following, the analytic themes presented in Table 2 will be discussed. The discussion aims to highlight opportunities and challenges in the use of VC in recovery in mental health, assessment, and therapy, and to identify for whom VC worked or did not work in the short-term care pathway from the therapists’ perspective. 

### “It’s Better Than Nothing”: Video Promotes Continuity and Access to Service

There has been great concern regarding the effect that the COVID-19 restrictions, societal lockdown, and resultant social isolation will have on mental health, particularly with regard to individuals who already have mental health problems or are in a recovery process [40,41]. The use of VC enables access to mental health services, and our findings show that VC does contribute to the overall realization of the continuity and maintenance of the therapist–service user relationship [4,8,20]. Nevertheless, some therapists experienced the initiation of a relationship online to be challenging, and our findings indicate that VC cannot perfectly replace regular face-to-face meetings. This is mainly due to poor clinical quality and technical challenges, as shown in previous research [8,42]. However, from the therapists’ perspective in this study, VC was found to help create trust and confidence before the first face-to-face meeting. In some instances, VC can even increase the involvement of the service user and enhance the recovery process, similar to findings in pre-COVID-19 studies [24].

Maintaining the relationship via VC also appears to influence the identity of both the service user and the therapists. For the service user, VC may reinforce the equation of the service user with their diagnosis, which may subsume their humanity entirely in the eyes of another [38]. For the therapists, however, it is also possible that the power balance between the therapists and the service user, and the perception of closeness and distance in their relationship may shift, especially if the therapist reflects on their own insecurity when using VC. Nevertheless, interaction on the screen may also increase the service users’ involvement in their own recovery process [8,26]. This may empower the service user if they are confident in coping with the technology, which may in turn further facilitate the recovery process.

### Life on the Screen: VC Affects the Therapists’ Work Situation

Our findings suggest that the working conditions for therapists can change for the worse when performing VC and might cause more stress in the work situation [43]. The therapists found VC to be more exhausting than face-to-face meetings, as staring into the screen required concentration and demanded a different kind of presence than being together in the same room. As such, the consequences of implementing video technology may, in the long-term, lead to burnout for the therapists, followed by an increase in sick leave [43]. Moreover, challenges may emerge when scaling up the services after a pilot phase [32]; these may include ensuring sufficient time between each consultation on video, and that all therapists are appropriately technologically equipped [44,45]. With regard to the practical aspects of conducting an effective VC, the therapists found it especially problematic when the technology failed or worked poorly. This often interrupted the flow of communication and hampered the therapists’ efforts to foster a safe and trusting environment. The therapists expressed concern that the use of VC may be challenging for the service user and lead to a worsening of their situation. Although close relationships and support from the service user’s family may be an important part of recovery [19], for others, relationships may negatively influence the recovery process [46,47]. Service users exposed to mental abuse or mistreatment in their home environment may need an alternative to home treatment through video [19]. Similarly, children are often dependent on their parents or next of kin as facilitators when offered consultations on video [48]. 

### Clinical Challenges When Using Video in Consultations

VC seems to be a workable alternative for following up with service users with less severe mental health problems; thus, depending on the service user’s specific context and state of mental health, the use of VC may be included in the process where appropriate. The therapists may also speak with the service users about which topics are suitable for VC to determine whether there are topics that should be avoided, including topics that may be too emotional for the service user to cope with without a face-to-face follow-up. Moreover, for a service user who is in personal recovery from severe mental problems, it may be important to be able to choose the topic of conversation, and to know that professional help is available even during a societal lockdown.

VC appears to be less appropriate both for those in need of long-term help and for mapping interactions in social relationships. With regard to the latter, a crucial part of providing mental health services is professional accountability, in which clinical judgment is an important part of mapping the patient’s condition to assess their needs and at which level to provide services. In this context, VC can be perceived as a filter that can obscure emotions and make it more difficult to evaluate service users’ overall mental conditions [4]. This challenges the quality of the therapists’ clinical judgment [49], which is at the core of the therapists’ professional practice, and how they see and speak to the patient. The technology itself may make it difficult to provide effective and quality care, which in turn may challenge the relationship between the therapist and service user, and the therapist’s ability to follow up with the service user appropriately [50].

### The Future of Video Consultations in Mental Health Care

The progression of mental health care requires new ways of providing continuous follow-up in different formats based on changes in the service users’ condition and circumstances. A variety of consultation models—face-to-face meetings, video consultations, home care, cocreation meetings, and even in-hospital treatment—may be necessary to provide appropriate care. Based on an analysis of media coverage during the COVID-19 lockdown, Idland [51] argues that although VC will be used as a supplement to face-to-face consultations in the future, most people will still return to face-to-face meetings as soon as possible. The change in the numbers of VCs performed in the hospital under study reflect a similar trend: 74% of the consultations took place via video in the second part of March 2020, followed by a decrease to 21% when the restrictions were eased in May. These numbers (Table 1) are not sufficient to draw conclusions due to the short time period, but they are an indication that can be used for reflection toward the future use of VC in mental health care. The findings clearly indicate that some therapists and service users did not find VC satisfying or safe enough to replace face-to-face consultations in the long run. Further studies are needed to investigate how the use of VC can be perceived as safe and satisfying in normal situations. 

### Conclusions

The COVID-19 restrictions forced rapid changes in the organization of hospitals and in the treatment of different conditions in the field of mental health. This situation may represent the start of a permanent change in the way mental health services are provided. Indeed, similar changes are already recommended (and sometimes required) by the World Health Organization [52], based on a growing population struggling with mental health problems and increasing challenges regarding how to treat and reach out to those who need help. This study of therapists’ experiences with VC in a mental health hospital in Norway during COVID-19 restrictions indicates that there are overall advantages to using VC when circumstances do not permit face-to-face consultations. Although the continuity that VC offers was seen as a valuable asset, the quality of the therapy was considered to be poorer on video than in face-to-face meetings. Various negative aspects related to the therapeutic environment such as lack of safety for the most vulnerable service users and topics unsuitable for VC lowered the therapists’ overall impression of the service.

Using VC in therapy may offer opportunities for empowerment by letting the service user select VC as a medium, and may make the service more accessible and available despite physical challenges such as immobility. Access to VC is especially important considering the societal impact of COVID-19. Meeting digitally provides the opportunity to follow up with and take care of the service user’s needs. A range of potential advantages appear when transferring parts of the mental health services into digital services and increasing the use of VC, including increased number of service users in treatment, increased satisfaction of both service users and therapists, improved outcomes, destigmatization, and more direct time expenditure on care by the therapists.

### Strengths and Limitations of the Study and Issues for Further Research

This study was performed within the context of COVID-19 restrictions, during which the entire department was required to use VC to maintain the treatment of service users in recovery. This allowed access to therapists with both negative and positive perceptions and experiences of digital communication therapy, avoiding the challenge of biased data from digital pioneers. A potential weakness of the study is its reliance on digital interviews. As demonstrated in this study, communicating via video can create a filter and a distance between the actors involved; as such, the information derived from the interviews may have been different if the interviews had been conducted face-to-face.

There is a need for further investigation, including qualitative research, to build solid and evidence-based knowledge that can contribute to developing tailored services for people in recovery and in need of mental health care. Further research should focus on service users’ experiences; cocreation between different stakeholders; and how to scale up the use of VC while ensuring that the service provided is appropriate, safe, and available.

## Abbreviations

eHealth: electronic health
EPJ: electronic patient journal
VC: video consultation
